# Binary Solvents Dispersive Liquid—Liquid Microextraction (BS-DLLME) Method for Determination of Tramadol in Urine Using High-Performance Liquid Chromatography

**DOI:** 10.1186/2008-2231-22-25

**Published:** 2014-02-03

**Authors:** Vahid Kiarostami, Mohamad-Reza Rouini, Razieh Mohammadian, Hoda Lavasani, Mehri Ghazaghi

**Affiliations:** 1 Department of Chemistry, North Tehran Branch, Islamic Azad University, P.O. Box 1913674711, Tehran, Iran; 2 Biopharmaceutics and Pharmacokinetics Division, Department of Pharmaceutics, Faculty of Pharmacy, Tehran University of Medical Sciences, Tehran, Iran; 3 Department of Applied Chemistry, Faculty of Science, Semnan University, Semnan, Iran

**Keywords:** Dispersive liquid-liquid microextraction, Tramadol, HPLC, Urine

## Abstract

**Background:**

Tramadol is an opioid, synthetic analog of codeine and has been used for the treatment of acute or chronic pain may be abused. In this work, a developed Dispersive liquid liquid microextraction (DLLME) as binary solvents-based dispersive liquid-liquid microextraction (BS-DLLME) combined with high performance liquid chromatography (HPLC) with fluorescence detection (FD) was employed for determination of tramadol in the urine samples. This procedure involves the use of an appropriate mixture of binary extraction solvents (70 μL CHCl3 and 30 μL ethyl acetate) and disperser solvent (600 μL acetone) for the formation of cloudy solution in 5 ml urine sample comprising tramadol and NaCl (7.5%, w/v). After centrifuging, the small droplets of extraction solvents were precipitated. In the final step, the HPLC with fluorescence detection was used for determination of tramadol in the precipitated phase.

**Results:**

Various factors on the efficiency of the proposed procedure were investigated and optimized. The detection limit (S/N = 3) and quantification limit (S/N = 10) were found 0.2 and 0.9 μg/L, respectively. The relative standard deviations (RSD) for the extraction of 30 μg L of tramadol was found 4.1% (n = 6). The relative recoveries of tramadol from urine samples at spiking levels of 10, 30 and 60 μg/L were in the range of 95.6 – 99.6%.

**Conclusions:**

Compared with other methods, this method provides good figures of merit such as good repeatability, high extraction efficiency, short analysis time, simple procedure and can be used as microextraction technique for routine analysis in clinical laboratories.

## Background

Tramadol ((±) cis-2-[(dimethylamino) methyl]-1-(3methoxyphenyl) cyclohexanol hydrochloride) is an opioid, synthetic analog of codeine and is not currently classified as a controlled substance [1-3]. Tramadol has been used since 1977 like other narcotics applied for the treatment of acute or chronic pain may be abused [4]. In Iran, this drug is easily available for patient without prescription and according to annual reports of ministry of health; 350 million tramadol tablets (100 mg) were sold in 2006–2007 and recently, it has become one of the most widely dispensed analgesics in Iran’s essential drugs list [5,6]. The extraction of tramadol from biological samples has usually been carried out by using liquid-liquid extraction (LLE) and solid phase extraction (SPE) [4,7-9]. However, LLE is time consuming and requires large amounts of organic solvent and SPE uses much less than LLE, but can be relatively expensive. Recently, other extraction methods as free solvent and miniaturized extractions, such as liquid phase microextraction (LPME) [10,11], solid phase microextraction (SPME) [12], solvent bar microextraction (SBME) [13], liquid phase microextraction with back extraction (LPME-BS) [14], three - phase hollow fiber liquid phase microextraction (HF-LPME) [15], have successfully been developed for determination of tramadol from different matrices. Dispersive liquid-liquid microextraction (DLLME) is a miniaturized liquid extraction that was introduced in 2006 by Rezaee and coworkers [16]. However, in this method, the selection of extraction solvents is limited to one type of heavier or lighter extraction solvent than water.

In our previous work, a new method based on DLLME methodology as binary solvents–based dispersive liquid-liquid microextraction (BS-DLLME) was developed for determination of patulin from apple juice samples [17]. In this method, two kinds of extraction solvents (mixture of low and high density solvents) can be used simultaneously. In the present study, a rapid, sensitive and simple BS-DLLME and high performance liquid chromatography coupled with florescence detection has been carried out for the extraction and pre-concentration of tramadol in urine samples.

## Methods

### Reagent

HPLC-grade methanol, acetonitrile, acetone, chloroform, analytical grade ethyl acetate and deionized water were obtained from Merck chemical co (Darmstadt, Germany). Carbon tetrachloride (CCl_4_) with grade of trace analysis was obtained from Merck. The pure substances of tramadol were kindly gifted by Grünenthal chemical co (Stolberg, Germany). Phosphoric acid, sodium hydroxide and sodium chloride were all of analytical grade from Merck and were used without further purification. All the glassware used in experiments first washed with HPLC grade water and acetone and then dried in an oven. For calibration studies, blank urine samples were kindly obtained from one female healthy volunteer in our lab which not exposed to the mentioned drug. The use of tramadol in healthy subjects has been approved in Tehran University of Medical Sciences ethics committee (Ethics board code 4233).

For recovery studies, fresh urine samples from three male volunteers with abuse of tramadol were kindly provided by the Loghman hospital (Tehran, Iran). The study of tramadol pharmacokinetics in drug abused subjects has been approved in Tehran University of Medical Sciences ethics committee (Ethics board code 20324).

Standard solution of tramadol in concentration of 1 mg/mL was prepared by dissolving 10 mg of this compound in 10 mL HPLC-grade water. Working standard solutions of tramadol were prepared by dilution of the stock solution using HPLC-grade water. Stock and standard solutions of this compound were stored at 4°C.

#### Instrumentation

For separating and analyzing the drug a WellChrom HPLC instrument from Knauer Company (Berlin, Germany) was applied. The chromatographic apparatus equipped with a fluorescence RF-10AXL detector (excitation wavelength of 200 nm and emission wavelengths of 301 nm). Gradient HPLC K-1001 pump and online K-5020 degasser. A Rheodyne model 7725i injector with a 20 μL loop was applied to inject the samples. Chromatographic data were acquired and analyzed using ChromGate Chromatography Software from Knauer Company. Separation was carried out on a ChromolithTM Performance RP-18e, 100 mm × 4.6 mm column (Merck, Darmstadt, Germany) protected by a ChromolithTM Guard Cartridge RP-18e 5 mm × 4.6 mm. A mixture of water and methanol (81:19 v/v) adjusted to pH 2.5 by phosphoric acid at a flow rate of 2 mL/min in isocratic elution mode was used as a mobile phase. Eppendorf centrifuge 4515c (Netheler-Hinz GMBH Germany) was used for sedimentation of the dispersive phase.

#### Sample preparation

Standard solutions containing 100 μg/mL of tramadol was prepared in HPLC grade water at (4°C) and brought to room temperature just prior to use. The pH of urine sample containing 100 μg/mL tramadol (spiked urine sample) at pH = 10 by addition of 5 M NaOH. Then 5 mL of this sample was placed in centrifuge tube, after centrifugation at 1133 × g for 10 min, upper solution was separated from sediment and was transferred to a 10 mL glass test tube [18].

##### BS-DLLME procedure

A mixture of a disperser solvent and binary extraction solvents (ethyl acetate and chloroform) were injected rapidly into the mentioned pretreated urine solution (5 ml) by 1.00 mL syringe and immediately a cloudy solution was formed. After centrifuging the cloudy solution for 10 min at 1133 × g, the dispersed fine droplets of ethyl acetate and chloroform were settled in the bottom of conical test tube. The deposited phase was transferred to another glass tube and evaporated to dryness under a gentle air stream. The residue was dissolved in 70 μL mobile phase and 20 μL was injected into the HPLC with fluorescence detection system for analysis.

## Results and Discussion

In order to optimize the BS-DLLME for determination of tramadol in urine samples, the effective parameters on extraction efficiency such as the type and volume of high density extraction solvent, the volume of ethyl acetate as low density extraction solvent, the type and volume of disperser solvent, salt addition and extraction time were studied. Statistical calculations (single factor analysis of variance and unpaired t-test) carried out with Microsoft® excel 2007 for comparing of data. Significant difference was pretended if the probability level (p) was less than of 0.05.

### Selection of high density extraction solvent

**Figure 1:**
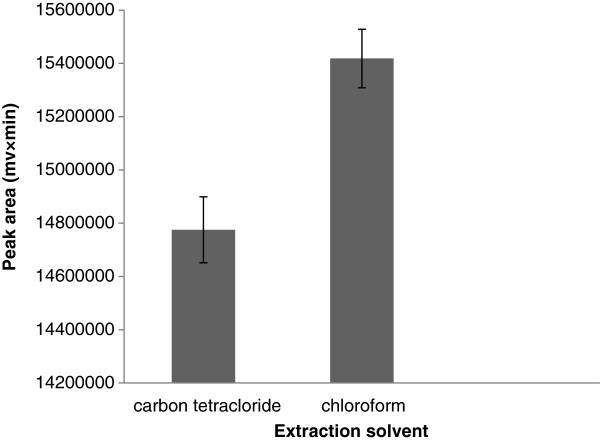
**Effect of high density extraction solvent type on the extraction efficiency.** Extraction condition: tramadol concentration, 100 μg/ L; volume of extraction solvent, 50 μL; disperser solvent and its volume, 0.6 mL acetonitrile; no salt addition.

Selection of high density extraction solvent is very important in BS-DLLME procedure. For this purpose chloroform (CHCl_3_, density 1.49 g/L), carbon tetrachloride (CCl_4_, density 1.59 g/ml) and dichloromethane (CH_2_Cl_2_, density 1.32 gmL^−1^) selected in this study. A series of urine samples were surveyed by using 0.6 mL of acetone (as disperser solvent) contains 70 μL of different high density extraction solvents. In the presence of dichloromethane as extraction solvent, no droplet was formed and therefore ignored as extraction solvent. As shown in Figure 1, chloroform has better extraction recovery with significant effect (unpaired t-test assuming unequal variances, p < 0.05) than the other tested solvents. Therefore, CHCl_3_ was selected as the extraction solvent in subsequent experiments.

### Effect of extraction solvent volume

**Figure 2:**
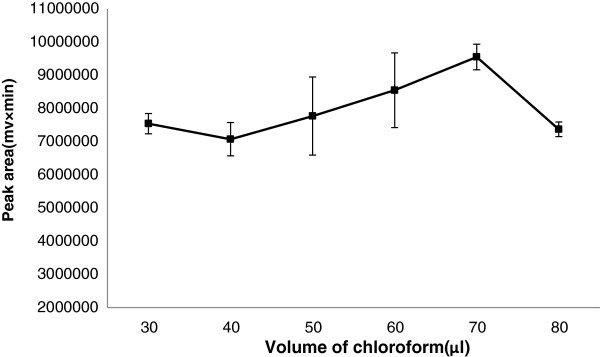
**Effect of volume of chloroform on the extraction efficiency.** Extraction condition: tramadol concentration, 100 μg/L; disperser solvent and its volume, 0.6 mL acetonitrile; extraction solvent, chloroform; no salt addition.

To examine the effect of high density extraction solvent volume on performance of the presented BS-DLLME procedure, we used acetone with a constant volume (0.6 mL) and different volume of CHCl_3_ (30–80 μL). As indicated in Figure 2, the extraction efficiency increases with CHCl_3_ volume from 40 to 70 μL. Above 70 μL of chloroform, the recovery decreases probably due to decrease in the number of droplets available for extraction. According to these results, 70 μL of chloroform with remarkable effect (single factor analysis of variance, p < 0.05) was selected as the optimal extraction solvent volume in the BS-DLLME procedure.

### Effect of ethyl acetate and its volume

**Figure 3:**
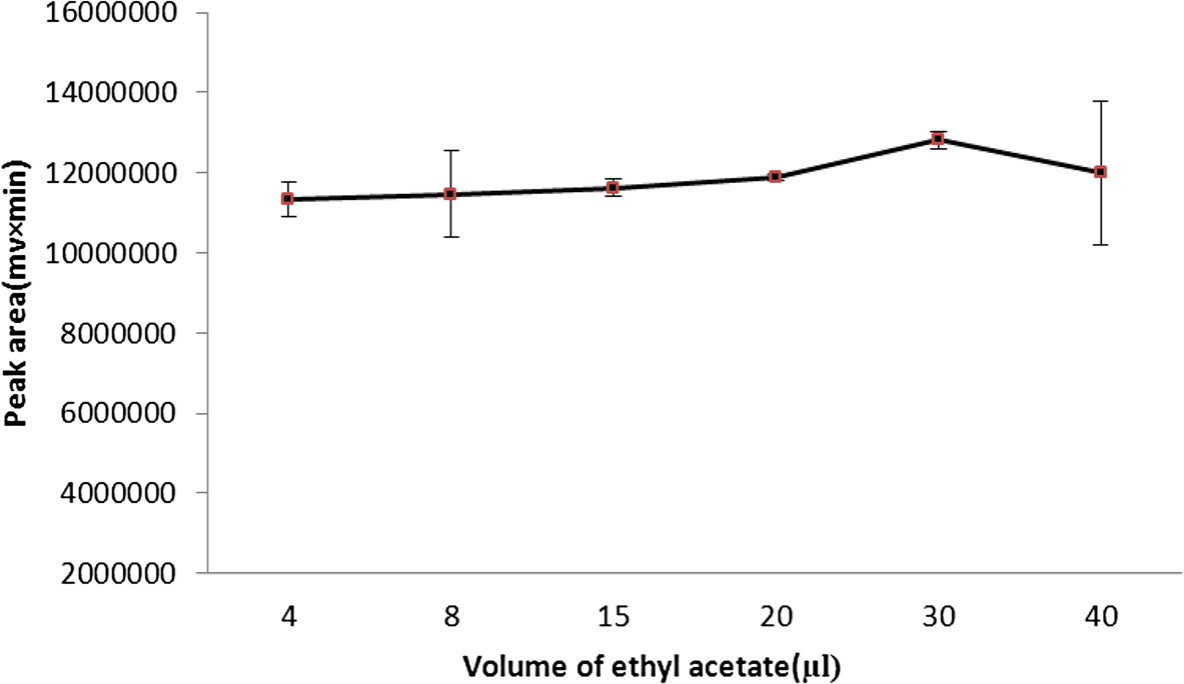
**Effect of ethyl acetate volume on the extraction efficiency.** Extraction condition: tramadol concentration, 100 μg/ L; extraction solvent and its volume, 70 μL chloroform; disperser solvent and its volume, 0.6 mL acetonitrile; no salt addition.

The effect of ethyl acetate (C_4_H_8_O_2_ , density 0.897 g /ml) as lighter extraction solvent in BS-DLLME of tramadol in urine samples was studied. For considering the influence of ethyl acetate volume on extraction efficiency, different volumes of ethyl acetate (0 – 40 μL), fixed volumes of chloroform (70 μL) and acetonitrile (600 μL) were used for BS-DLLME procedure. As can be seen in Figure 3, initially the extraction efficiency increased and then decreased by increasing the volume of ethyl acetate. Thus 30 μL of ethyl acetate as lighter density extraction solvent with lower error bar and 70 μL of chloroform as higher density extraction solvent (100 μL of binary extraction solvents) were selected in the subsequent experiments.

### Selection of disperser solvent

**Figure 4:**
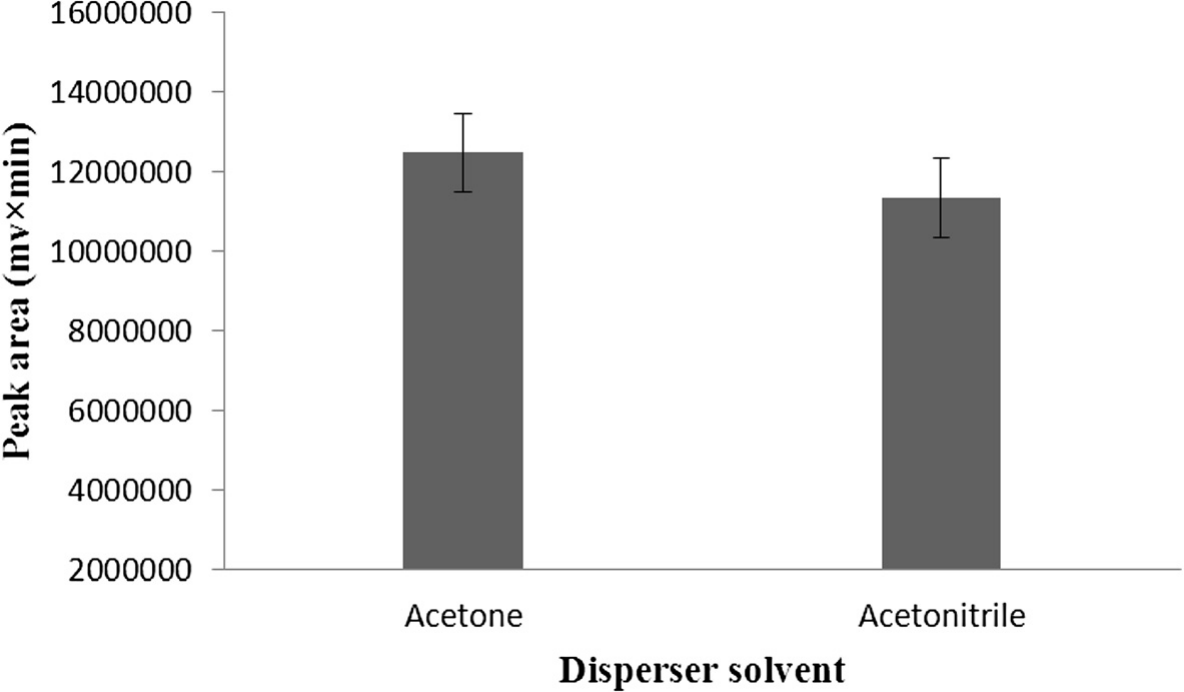
**Effect of disperser solvent type on the extraction efficiency.** Extraction condition: tramadol concentration, 100 μg/L; binary extraction solvents and their volume, 100 μL mixture of ethyl acetate and chloroform (3:7 v/v); disperser solvent volume, 0.6 mL; no salt addition.

The most important factor affecting the selection on disperser solvent is relative miscibility of the disperser solvent with the binary extraction solvents and aqueous phase. Methanol, ethanol, acetonitrile and acetone exhibit adequate properties and were studied as disperser solvents. Then, the effect of these solvents on performance of BS-DLLME was investigated. For these purpose, 600 μL of disperser solvent and 100 μL of binary extraction solvents were used. In ethanol and methanol, whitish sediment was formed, which cannot be separated from supernatant. Thus, the sediment is interfered for analysis and therefore methanol and ethanol were ignored as disperser solvents. According to Figure 4, acetone provided higher extraction efficiency with significant effect (one-tailed unpaired t-test, p < 0.05) and was chosen as disperser solvent in the following experiments.

### Effect of disperser solvent volume

**Figure 5:**
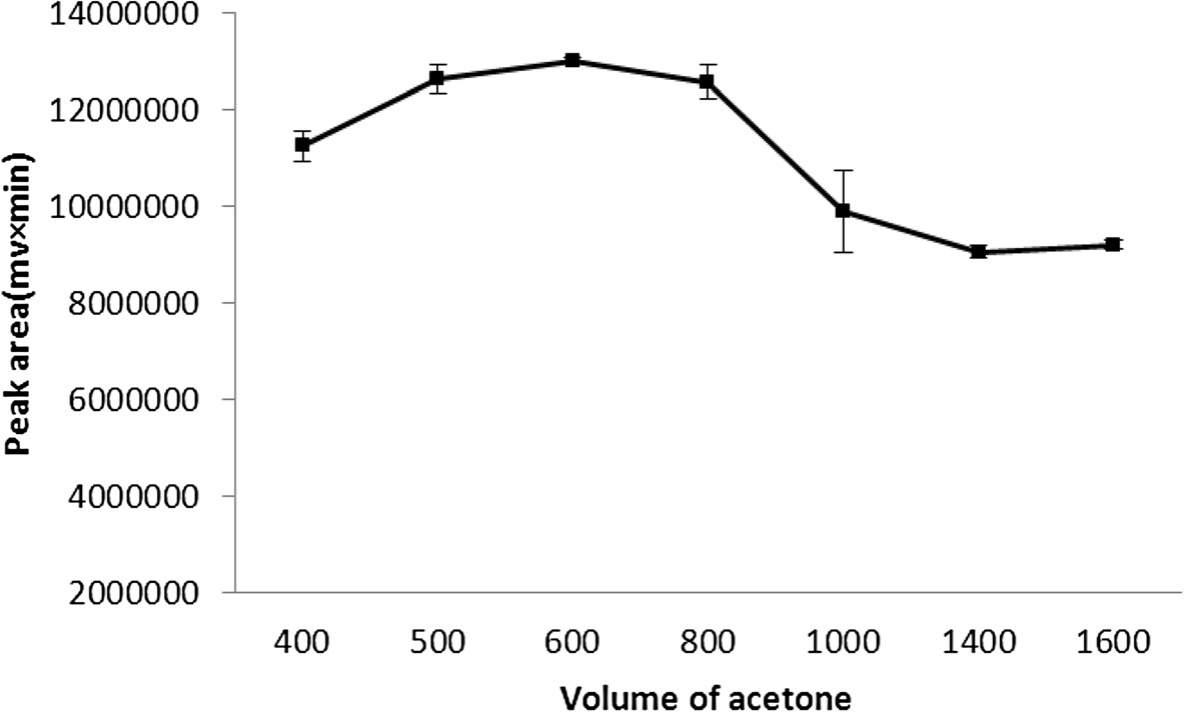
**Effect of disperser solvent volume on the extraction efficiency.** Extraction condition: tramadol concentration, 100 μg/ L; binary extraction solvents and their volume, 100 μL mixture of ethyl acetate and chloroform (3:7 v/v); disperser solvent, acetone; no salt addition.

Effect of acetone (as disperser solvent) volume surveyed by using various volumes of acetone (400 – 1600 μL), while all experimental condition were kept constant (100 μL binary extraction solvents). As shown in Figure 5, the extraction efficiency increased by increasing the acetone volumes from 400 μL 600 μL. Above 600 μL of acetone the extraction efficiency decreased for tramadol, probably due to increase of tramadol solubility in the higher volume of acetone. Therefore 600 μL of acetone with significant effect (single factor analysis of variance, p < 0.05) was selected as the optimal disperser solvent volume in BS-DLLME procedure.

### Effect of salt addition

**Figure 6:**
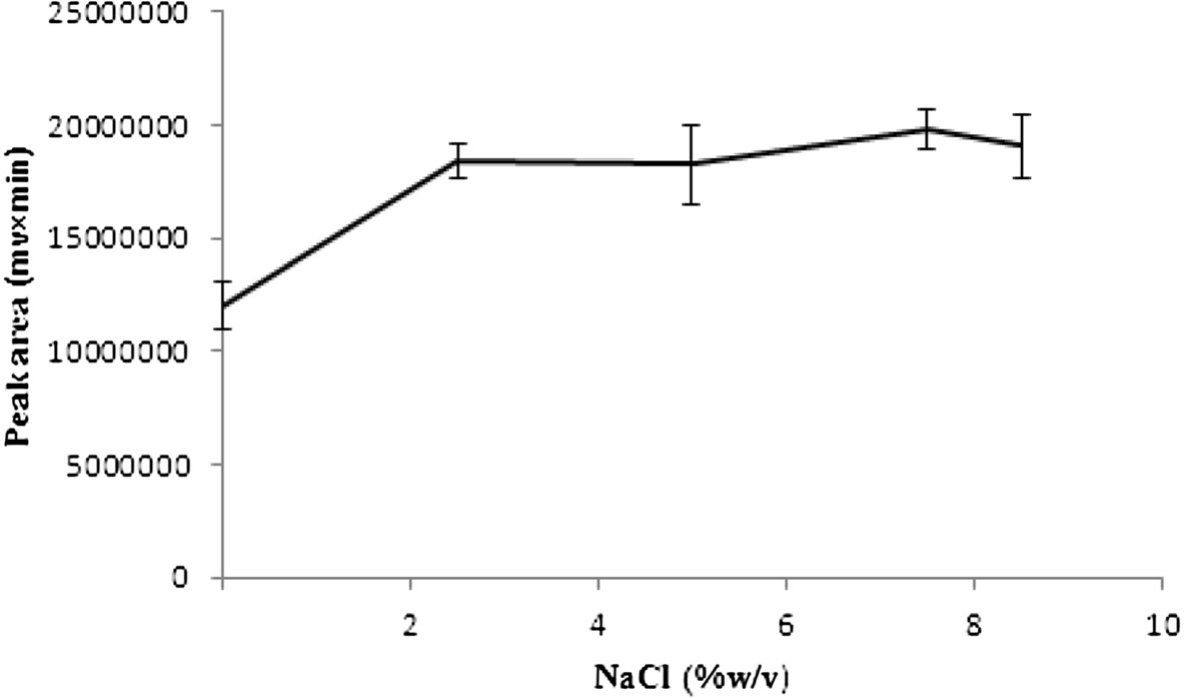
**Effect of salt addition on the extraction efficiency.** Extraction conditions: tramadol concentration, 100 μg/L; binary extraction solvents and their volume, 100 μL mixture of ethyl acetate and chloroform (3:7 v/v); disperser solvent and its volume, 0.6 mL acetone.

To evaluate the effect of salt addition on BS-DLLME performance, various experiments were performed by adding different amounts of NaCl (0 - 10%, w/v). Adding of salt may have different results in DLLME like increasing [19], reducing [20] and no impact on recovery [17]. In this study as indicated in Figure 6, by adding (0-10%, w/v) NaCl, initially, increasing the NaCl concentration to 7.5%, the extraction efficiency increased then decreased by increasing the NaCl concentration. Thus, 7.5% (w/v) NaCl with significant effect (single factor analysis of variance, p < 0.05) was selected for further experiments.

### Effect of extraction time

**Figure 7:**
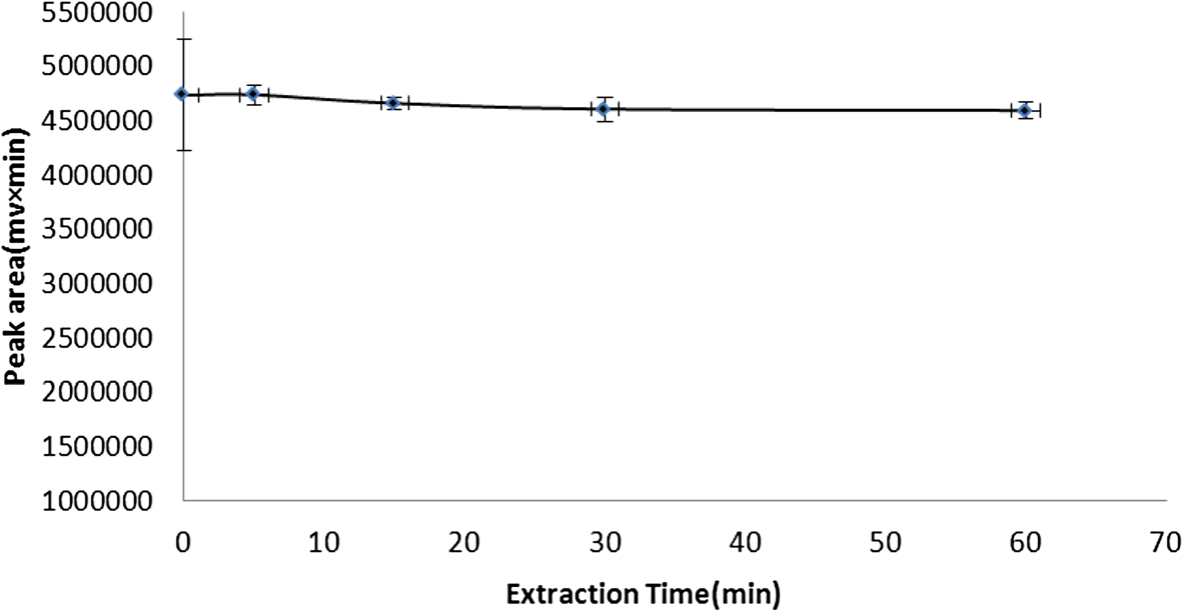
**Effect of extraction time on the extraction efficiency.** Extraction conditions: tramadol concentration, 100 μg/L; binary extraction solvents and their volume, 100 μL mixture of ethyl acetate and chloroform (3:7 v/v); disperser solvent and its volume, 0.6 mL acetone; 7.5% NaCl.

In DLLME, extraction time is defined as the interval between injection of the mixture of organic solvents and centrifugation [21]. Generally extraction time has no impact on DLLME procedure [22]. As shown in Figure 7, the effect of extraction time on the extraction efficiency in the range of 0 – 60 min was investigated. The results showed that, the extraction time had no remarkable effect (single factor analysis of variance, p > 0.05) on BS-DLLME procedure.

Overall, according to optimum condition, the values of studied factors were as follows: 0.6 mL acetone as disperser solvent, 100 μL binary extraction solvents (70 μL of chloroform and 30 μL of ethyl acetate) and 7.5% (w/v) NaCl.

### Analytical performance

Under optimization condition using blank urine samples spiked at various concentration of tramadol, the characteristic calibration data were obtained. As can be seen in Table 1, linearity was observed for tramadol in the range of 1–130 ng/ml with the determination coefficient (r^2^) of 0.993. The enrichment factor for tramadol was obtained 71.42. The limit of quantification (LOQ, S/N = 10) and limit of detection (LOD, S/N = 3) were 0.9 and 0.2 ng/ml, respectively. The relative standard deviation (RSD) determined by six replicate experiments was 4.1 at a concentration of 30 ng/ml.

**Table 1:** Figures of merit in the BS-DLLME

**Analyte**	**r**^**2, a**^	**Regression equation**	**RSD, %(N = 6)**	**LOD**^**b**^**/ng/ml**	**LOQ**^**c**^**/ng/ml**	**LDR**^**d**^**/ng/ml**
Tramadol	0.993	Y = 78382x + 24663	4.1	0.2	0.9	1-130

^a^Determination coefficient.

^b^Limit of detection.

^c^Limit of quantification.

^d^Linear dynamic range.

### Application in real sample

The applicability of proposed BS-DLLME method was evaluated for the analysis of different urine samples, which collected from the patient volunteers under treatment in Baharloo hospital (Tehran, Iran) without any dilution. The pH of urine samples was adjusted at 10 by dropwise addition of 5 M NaOH. The relative recoveries of tramadol from urine samples were determined at spiking level of 10, 30 and 60 ng/ml. As can be seen in Table 2, the results of six replicate experiments of each sample using the BS-DLLME method are in the range of 95.6 – 99.6%. Thus the proposed method can be applied for the determination of tramadol in urine samples.

**Table 2:** Relative recoveries of tramadol in urine samples

**Sample**	**Initial concentration**	**Concentration added/μg/L**	**Concentration determined mean ± SD**^**b**^**/μg/ L**	**Relative recovery**
Urine	nd^a^	10	9.96 ± 0.2	99.6
		30	28.67 ± 1.5	95.6
		60	59.03 ± 3.48	98.4

Extraction conditions: binary extraction solvents and their volume, 100 μL mixture of ethyl acetate and chloroform (3:7 v/v); disperser solvent and its volume, 0.6 mL acetone; 7.5% NaCl.

^a^Not detected.

^b^Standard deviation.

**Figure 8:**
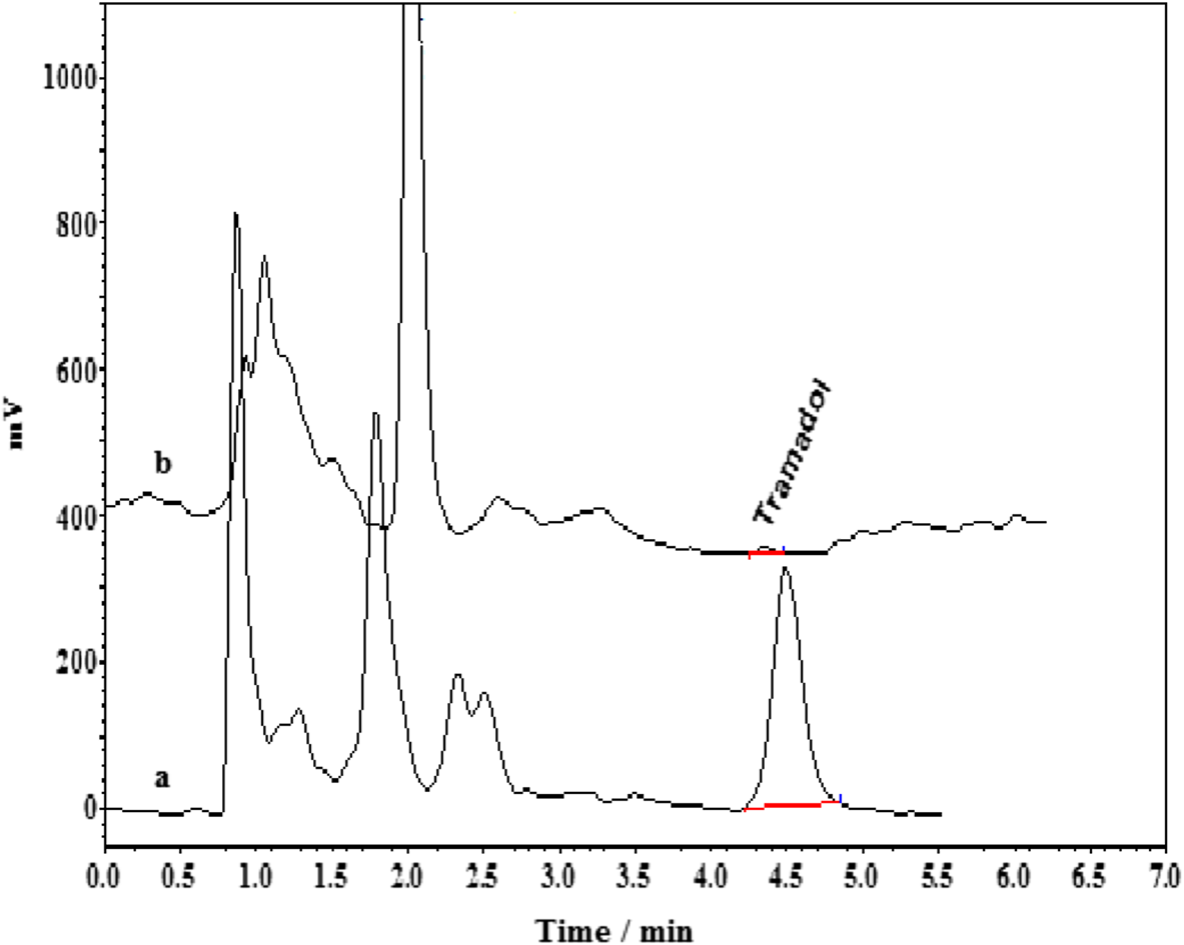
**Chromatograms for the analysis of tramadol in urine samples. (a)** HPLC chromatogram of the urine sample (volunteer with abuse of tramadol) spiked with tramadol at concentration level 60 μg/ Lafter employing BS-DLLME, **(b)** HPLC chromatogram of blank human urine sample (healthy volunteer) after performing BS-DLLME. Extraction conditions: tramadol concentration, 100 μg/L; binary extraction solvents and their volume, 100 μL mixture of ethyl acetate and chloroform (3:7 v/v); disperser solvent and its volume, 0.6 mL acetone; 7.5% NaCl.

Representative chromatograms with good resolution obtained for tramadol, from blank human urine sample (healthy volunteer) and urine sample (volunteer with abuse of tramadol) spiked with tramadol under the optimum BS-DLLME condition are shown in Figure 8.

### Comparison of BS-DLLME with other methods

The figures of merit of BS-DLLME method for determination of tramadol in urine sample have been compared to earlier reported methods. As shown in Table 3, the proposed method has lower LOQ, good linear dynamic range (LDR) and higher relative recoveries in comparison to earlier methods. In addition, the extraction time in BS-DLLME is shorter and this method does not involve any labor-intensive and time consuming steps. Determination of tramadol at low levels in plasma is also important for the pharmacokinetic analysis [23] and therefore, DLLME procedure can be used as an alternative procedure for the previous analytical methods.

**Table 3:** Comparison of the proposed method with other methods for the extraction of tramadol

**Method**	**Sample matrix**	**RR**^**a**^**, %**	**LDR/μg/ L**	**LOD/μg/ L**	**LOQ/μg/ L**	**r**^**2**^	**Ref**^**e**^**.**
HPLC-FL^b^, HPLC-MS/MS	Dog urine	82	1-1000	5	10	0.999	[24]
Liquid extraction HPLC	Human plasma	74.7-80.8	2.5-500	-	2.5	0.997	[25]
SPE^c^/GC-MS	Human oral fluid	87.7	10-100	-	10	0.999	[9]
MISPE^d^/HPLC-UV	Human plasma, urine	>91	2-300	1.2	3.5	-	[4]
LLE-ion pair formation	Urine	-	10000-50000	400	1200	0.997	[26]
BS-DLLME/HPLC-FL	Human urine	95.6-99.6	1-130	0.2	0.9	0.993	This work

^a^Relative recovery.

^b^High performance liquid chromatography with fluorescence Detection.

^c^Solid-phase extraction.

^d^Molecularly imprinted solid-phase extraction.

^e^Refferences.

## Conclusions

The present study has proposed a new method for determination of tramadol in urine samples using the BS-DLLME coupled with HPLC-fluorescence detection. BS-DLLME method provides good repeatability and higher recoveries within a short time. The comparison of this method with others demonstrated that BS-DLLME was very fast, simple and inexpensive with good figures of merit. In comparison to conventional DLLME, the selection of extraction solvents in BS-DLLME is not limited to high density solvents. In summary, the developed methodology shows good performance of analytical protocol, exhibits excellent recoveries for tramadol in urine samples.

## Abbreviations

BS-DLLME: Binary solvents dispersive liquid liquid microextraction; SPE: Solid phase extraction; SPME: Solid phase microextraction; MISPE: Molecular imprinting solid phase extraction; FL: Fluorescence detection.

## Competing interests

The authors declare that have no competing interests.

## Authors’ contributions

VK supervised the project and prepared the manuscript, MRR gave consultation on pharmaceutical analysis and final manuscript preparation and made substantial contribution for performing the project, RM carried out the data analysis and helped to draft the manuscript, HL gave consultation on HPLC assay and MGH performed the data analysis. All authors read and approved the final manuscript.
